# High Therapeutic Efficiency of Magnetic Hyperthermia in Xenograft Models Achieved with Moderate Temperature Dosages in the Tumor Area

**DOI:** 10.1007/s11095-014-1417-0

**Published:** 2014-06-03

**Authors:** Susanne Kossatz, Robert Ludwig, Heidi Dähring, Volker Ettelt, Gabriella Rimkus, Marzia Marciello, Gorka Salas, Vijay Patel, Francisco J. Teran, Ingrid Hilger

**Affiliations:** 1 Institute for Diagnostic and Interventional Radiology I, Jena University Hospital – Friedrich Schiller University Jena, Bachstraße 18, 07740 Jena, Germany; 2 Instituto de Ciencia de Materiales de Madrid, ICMM-CSIC, Sor Juana Inés de la Cruz 3, Campus Universitario de Cantoblanco, 28049 Madrid, Spain; 3 Instituto Madrileño de Estudios Avanzados en Nanociencia, Campus Universitario de Cantoblanco, 28049 Madrid, Spain; 4 Liquids Research Limited, Deiniol Road, Bangor, Gwynedd UK; 5 Unidad Asociada de Nanobiotecnología CNB-CSIC & IMDEA Nanociencia, Campus Universitario de Cantoblanco, 28049 Madrid, Spain

**Keywords:** CEM43T90, *in vivo*, iron oxide nanoparticles, magnetic hyperthermia, temperature dose

## Abstract

**Purpose:**

Tumor cells can be effectively inactivated by heating mediated by magnetic nanoparticles. However, optimized nanomaterials to supply thermal stress inside the tumor remain to be identified. The present study investigates the therapeutic effects of magnetic hyperthermia induced by superparamagnetic iron oxide nanoparticles on breast (MDA-MB-231) and pancreatic cancer (BxPC-3) xenografts in mice *in vivo*.

**Methods:**

Superparamagnetic iron oxide nanoparticles, synthesized either *via* an aqueous (MF66; average core size 12 nm) or an organic route (OD15; average core size 15 nm) are analyzed in terms of their specific absorption rate (SAR), cell uptake and their effectivity in *in vivo* hyperthermia treatment.

**Results:**

Exceptionally high SAR values ranging from 658 ± 53 W*g_Fe_^−1^ for OD15 up to 900 ± 22 W*g_Fe_^−1^ for MF66 were determined in an alternating magnetic field (AMF, *H* = 15.4 kA*m^−1^ (19 mT), *f* = 435 kHz). Conversion of SAR values into system-independent intrinsic loss power (ILP, 6.4 ± 0.5 nH*m^2^*kg^−1^ (OD15) and 8.7 ± 0.2 nH*m^2^*kg^−1^ (MF66)) confirmed the markedly high heating potential compared to recently published data. Magnetic hyperthermia after intratumoral nanoparticle injection results in dramatically reduced tumor volume in both cancer models, although the applied temperature dosages measured as CEM43T90 (cumulative equivalent minutes at 43°C) are only between 1 and 24 min. Histological analysis of magnetic hyperthermia treated tumor tissue exhibit alterations in cell viability (apoptosis and necrosis) and show a decreased cell proliferation.

**Conclusions:**

Concluding, the studied magnetic nanoparticles lead to extensive cell death in human tumor xenografts and are considered suitable platforms for future hyperthermic studies.

**Electronic supplementary material:**

The online version of this article (doi:10.1007/s11095-014-1417-0) contains supplementary material, which is available to authorized users.

## INTRODUCTION

The treatment of tumors with heat has been proven to inhibit malignant proliferation and stimulate tumor cell death (1,2). Defined temperature regimes have been suggested to be necessary to effectively kill tumor cells. Two key studies in the mid-80s indicated that a thermal dose of 42°C for more than one hour will cause necrosis and/or apoptosis of cancer cells (3,4). It was shown that temperatures in the range between 39 and 42°C are effective for clinical hyperthermia (5,6) and that whole body hyperthermia at 39–41°C could significantly delay tumor growth in a xenotransplanted colon carcinoma model in rats (7). Subsequent preclinical and clinical studies however often suffered from difficulties to deliver therapeutic temperatures to tumor tissue and monitor the achieved temperatures, making it difficult to correlate the applied thermal dose with the respective therapy outcome (6,8,9). One reason for the, so far, non-satisfactory therapy outcomes is due to the lack of adequate temperature deposition right in the tumor tissue, particularly by utilization of whole body hyperthermia or external heating sources, such as ultrasound, microwave or infrared radiation (10). Increased side effects like burns on healthy tissue are further reasons why the benefits of hyperthermia have not been well established in the clinical routine.

An important parameter for the success of hyperthermia treatment is the intratumoral temperature distribution, which is measured by T90 values and which describes the temperature achieved or exceeded in 90% of the tumor area, together with the cumulative equivalent minutes at a T90 temperature of 43°C (CEM43T90) (4) as a measure of the temperature dose. In this context the temperature dose is a superior indicator for the therapy outcome to the temperature alone (11). In two important clinical trials, one in humans with superficial tumors and one in canines with soft tissue sarcoma, 20–100 CEM43T90 resulted in a better clinical outcome than 0.1–5 CEM43T90 (12,13). However, in the majority of clinical studies it became obvious that temperature dosages of 30–60 min CEM43 higher than 43°C were rarely achievable (6).

In contrast, the development of superparamagnetic nanoparticles (MNP) opened the window to locally deliver into tumor tissue therapeutic temperatures in the hyperthermic range. After selective and local accumulation in the tumor area, MNP act as heating sources when subjected to alternating magnetic fields. Hereto, heat loss mechanisms during magnetization reversal lead to a local temperature rise which can be controlled by modulating the field strength or frequency as well as the MNP structural features and mass.

The main advantage of MNP based targeting is the potential to localize the treatment right into the tumor area by a selective activation of the heating process when applying an alternating magnetic field. In this context, the heat power generated by MNP is dependent on their specific absorption rate (SAR) and the influence of the intracellular degradation and aggregation on preserving SAR values.

Due to the suitable benefits of magnetic hyperthermia on cancer treatment, we hypothesize that MNP with high SAR values and carefully controlled temperature dosages can be used to achieve efficient hyperthermia treatments *in vivo* compared to other methods, where the heat is externally delivered to the tumor.

Here, we sought to check this hypothesis using two subcutaneous xenograft models of breast and pancreatic cancer to identify cell line specific differences in treatment response. With the aim to assess the influence of physicochemical MNP properties on the induction of intratumoral heating and the resulting therapeutic effects we investigated the therapy outcome in course of hyperthermia treatment with two dispersions of superparamagnetic particles prepared by different strategies, whose heating potential was analyzed in conjunction with hyperthermia effects. Furthermore, we were seeking to identify correlations between temperature distribution, temperature dose and the tumor volume and corresponding histological parameters to gain further insight into the complex process of tumor therapy by magnetic hyperthermia.

## MATERIALS AND METHODS

### Synthesis of Nanoparticles

To obtain information on the differences in hyperthermic properties of MNP resulting from different synthesis routes, we have synthesized superparamagnetic iron oxide nanoparticles by two different chemical routes: OD15 MNP *via* organic route and MF66 MNP *via* aqueous route.

The synthesis of OD15 was done according to the protocol of Salas *et al.* 2012 (14) starting with an iron oleate complex. Iron(III)oleate was mixed under nitrogen with oleic acid in 1-octadecene. The mixture was warmed up with a heating mantle and initially stirred (100 rpm) until *T* = 60°C. Then stirring was stopped and the reaction heated to reflux (315°C) in order to narrow the MNP size distribution, enhance crystalline MNP and consequently to improve their magneto-thermal properties (14). After cooling to room temperature, the mixture was washed with ethanol, centrifuged, and the nanoparticles were magnetically separated. MF66 nanoparticles were produced by means of the co-precipitation technique (15).

The MNP were initially coated with oleic acid and dispersed in toluene and finally transferred to a solution of dimercaptosuccinic acid (DMSA) in dimethyl sulfoxide (DMSO). The coating with DMSA was followed by resuspension in distilled water, pH adjustment and sterile filtration (14). Nanoparticle size and shape were examined by transmission electron microscopy (TEM; 200 keV Microscope JEOL JEM 2000 FXII). The mean particle size and distribution were evaluated by measuring at least 200 nanoparticles and fitting the data to a log-normal distribution. The hydrodynamic diameter was determined *via* dynamic light scattering (DLS) and expressed as the Z-average size (Zetasizer Nano ZS, Malvern Instruments, Herrenberg, Germany) after nanoparticle suspension in water. Additionally, ζ-potential of the MNP was measured (Zetasizer Nano ZS, Malvern Instruments, Herrenberg, Germany).

### Specific Absorption Rate

The specific absorption rate (SAR) of the MNP was determined by measuring the temperature increase per unit of mass of MNP dispersion (200 μl) under given alternating magnetic field (AMF) conditions (*H* = 15.4 kA*m^−1^, *f* = 435 kHz). The MNP dispersion temperature was measured by using a commercial fiber optic temperature thermometer (TS5 & FOTEMPMK-19, Optocon AG, Dresden, Germany) with an experimental error of ±0.2°C.

The SAR values were determined by extracting the temperature increase immediately after switching on the alternating magnetic field (AMF) (16). The SAR determination is based on the equation:

$$ SAR=c*\frac{m_F}{m_P}*\frac{\Delta T}{\Delta t}, $$

where c is the specific heat capacity of the MNP dispersion, m_F_ the mass of the fluid, m_P_ the mass of the nanoparticles and ∆T/∆t the maximum value of the linear slope at initial times after switching on the alternating magnetic field.

To modulate particle immobilization which inhibits Brownian motion, as it occurs in tissue *in vivo*, MNP were dispersed in agarose gels (1% w/v) and in polyvinylalcohol hydrogels (PVA, 10% w/v) according to Gleichmar et al. 2009 and Ohta et al. 2004 (17,18). The polymerization time for agarose gels was 24 h at 8°C, at −20°C for hydrogels, respectively. The mass of the nanoparticle suspensions in water, agarose and hydrogels were determined right before the SAR measurements.

In order to compare heating efficiencies from measurements carried out in different MNP, laboratories, field strengths and frequencies, we have calculated the intrinsic loss power (ILP) of the studied MNP. According to Kallumadil *et al.* 2009, the ILP is a system-independent parameter, which allows comparison between SAR values obtained under different AC field strength and frequency conditions (19). For calculations, the following equation was used:

$$ ILP=\frac{ SAR}{H^2f}, $$

where H is the applied field strength and f the used frequency.

### Assessing Nanoparticle Internalization into Cells

To visualize the internalization of the MNP into MDA-MB-231 cells, 8,000 cells/well were seeded onto 8-well culture slides (BD Biosciences, Bedford, MA, USA), cultured overnight (37°C, 5% CO_2_) and afterwards incubated with 125 pg Fe/cell of the respective MNP formulation (OD15, MF66) for 24 h. Non-internalized MNP were removed by washing the cells three times with HBSS before fixation of cells with 4% (w/v) formaldehyde (10 min at 4°C). After two additional washing steps the iron was stained using the Prussian Blue staining method. To this end, slides containing fixed MDA-MB-231 cells were incubated in a 10% potassium ferrocyanide solution (Sigma-Aldrich, Steinheim, Germany) followed by a mix of 20% hydrochloric acid (Sigma-Aldrich, Steinheim, Germany) and 10% potassium ferrocyanide solution. Slides were covered with Faramount (DAKO, Glostrup, Denmark). Furthermore, to investigate thermosensitivity of MDA-MB-231 and BxPC-3 cells, cell viability with and without hyperthermia treatment was tested using the AlamarBlue assay (for details see Supplements).

### Xenograft Models and Tumor Implantation

All experiments were in accordance with international guidelines on the ethical use of animals and were approved by the regional animal care committee (reg. number 02-069/11). Throughout all procedures animals were anesthetized with 2.5% isoflurane (Aktavis, Langenfeld, Germany).

Subcutaneous tumors were induced on female athymic nude mice (Hsd:Athymic Nude-Foxn1^nu^) (Harlan Laboratories, Venray, The Netherlands). Hereto, 120 μl of Matrigel™ (BD Biosciences, Bedford, USA) containing 2 million MDA-MB-231 cells (human breast adenocarcinoma, doubling time *in vitro*: 1.8 ± 0.5 days) or BxPC-3 cells (human pancreatic adenocarcinoma, doubling time *in vitro*: 1.5 ± 0.3 days) (both ATCC, Wesel, Germany) were subcutaneously implanted. The experiments were started once tumors reached volumes between 80 and 500 mm^3^ as calculated by the formula V = π/6 × (length × width × height of the tumor) (20).

For each tumor model (breast and pancreatic tumors) four independent animal groups were used. Group 1 received magnetic hyperthermia treatments, which consisted in the MNP intratumoral injection and the exposure to an alternating magnetic field (AMF, *H* = 15.4 kA*m^−1^, *f* = 435 kHz) for 60 min. To investigate the impact of MNP alone on tumor growth, group 2 received only MNP, but no AMF treatment. Group 3 received ddH_2_O instead of MNP and was exposed to the AMF (*H* = 15.4 kA*m^−1^, *f* = 435 kHz) to investigate, if the AMF alone induced distinct effects on tumor growth. To monitor the treatment independent tumor growth, group 4 received only ddH_2_O.

### Application of the Magnetic Material

**Fig. 1:**
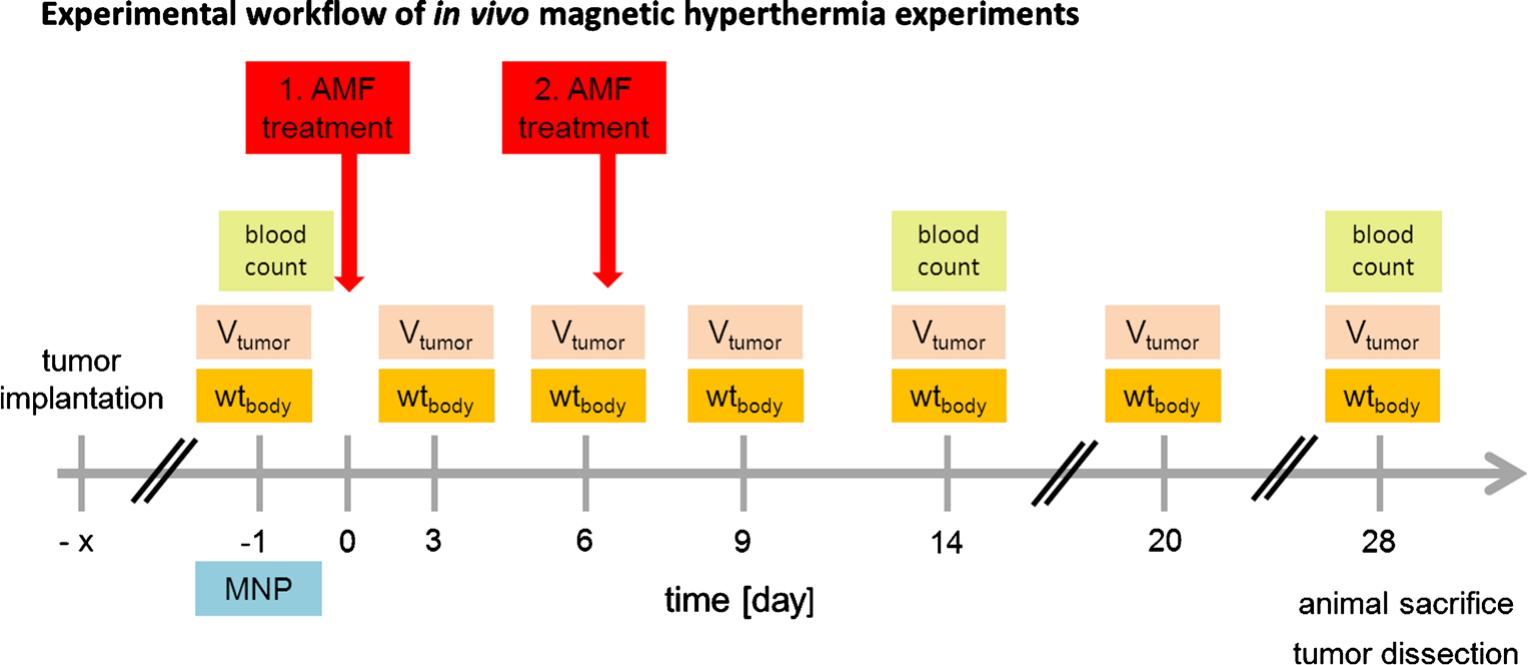
Experimental workflow of *in vivo* magnetic hyperthermia experiments. One and seven days after magnetic nanoparticle (MNP) application, magnetic hyperthermia was conducted on subcutaneous xenografts (AMF: *H* = 15.5 kA*m^−1^, *f* = 435 kHz) for 60 min. Tumor volume (V_tumor_) and body weight (wt_body_) were monitored at the indicated days. Blood count of the animals was determined on day 0, 14 and 28 post first magnetic hyperthermia. *AMF* alternating magnetic field.

The experimental workflow of the conducted *in vivo* magnetic hyperthermia experiments can be seen in Fig. 1. On day -1 mice of group 1 and group 2 received MNP. The day after, mice of group 1 and 3 were treated within the AMF. The BxPC-3-xenograft bearing mice of group 1 and 2 were injected intratumorally with 0.226 mg Fe per 100 mm^3^ (OD15:8.9 mg Fe/ml) or 0.087 mg Fe per 100 mm^3^ (MF66:4.3 mg Fe/ml). MDA-MB-231-xenograft bearing mice received 0.535 mg Fe per 100 mm^3^ (OD15) or 0.24 mg Fe per 100 mm^3^ (MF66) *via* intratumoral infiltration. Group 3 and 4 were injected using the same volume of ddH_2_O like the volume of MNP (group 1 and 2). Magnetic fluids and ddH_2_O were administered in a slow bolus into the tumor center by a stepwise backtracking of the needle. Injected volumes of MNP were chosen between 50 μl and 100 μl depending on the tumor entity.

### Magnetic Hyperthermia Treatments

For AMF exposure, mice of group 1 (magnetic hyperthermia) and group 3 (control, see above) were anaesthetized and placed in a water-bathed half pipe (38°C) to stabilize the body temperature during treatment. Tumors were selectively treated for 60 min under given AMF conditions (*H* = 15.4 kA*m^−1^, *f* = 435 kHz) (Fig. 1) and treatment was repeated at 7 days thereafter. During magnetic hyperthermia, tumor surface and rectal temperatures were monitored *in situ* using fiber optic temperature sensors (TS5 & FOTEMPMK-19, Optocon AG, Dresden, Germany). The tumor surface temperature was additionally monitored *in situ* using an infrared thermography camera (InfRec R300, Nippon Avionics Co., Ltd., Tokyo, Japan) during and 10 min pre- and post-treatment.

### Effects of Magnetic Hyperthermia on Tumor Volume and Blood Count

Tumor volume (caliper) and body weight were measured every 3 days (Fig. 1). The volume growth rate of untreated tumors for both xenograft models was calculated as the increase in tumor volume in percent per day related to the last measurement. Then the arithmetic mean of the tumor volume growth rates between all measurements was taken. On day -1, 14 and 28 after the initial treatment (Fig. 1), levels of red and white blood cells and hemoglobin were measured *via* hematology (Sysmex pocH-100i, Sysmex Deutschland GmbH, Norderstedt, Germany).

### Heat Maps

Heat maps (temperature distribution maps) of the treated tumor region were extracted from the surface temperature data recorded by infrared thermography. In this context and using the thermal images (wavelengths between 8 and 14 μm) taken at 10, 30, and 50 min after switching on the AMF, a polygonal region of interest (ROI, between 170 and 1,050 pixel) was placed over each tumor in a size specific manner (see Supplementary information). The temperature data of each pixel was extracted. T90 temperatures and cumulative equivalent minutes at 43°C (CEM43T90) were calculated according to Sapareto and Dewey 1984 (4). Additionally, the contribution of different tumor temperatures to the therapeutic effects was calculated more in detail. Accordingly, the temperature data were grouped in three distinctive categories: proportion of depicted tumor surface (in %) (I) with moderate hyperthermic temperatures (T < 43°C, (6)), (II) hyperthermic temperatures (T 43–45°C), and (III) mild thermoablative temperatures (T > 45°C). The relative contribution of each category to the whole tumor surface temperature was calculated, both AMF treatments (see Fig. 1) were summed up and plotted against the relative tumor volume 28 days after the initial magnetic hyperthermia.

### Hematoxylin & Eosin Staining

Formalin-fixed and paraffin-embedded xenografts were subjected to hematoxylin staining according to Mayer (Sigma-Aldrich, Steinheim, Germany), followed by an 0.1% eosin B staining (Sigma-Aldrich, Steinheim, Germany). After washing in increasing concentrations of ethanol (50%, 70% and 96% (w/v)) and xylol, slides were covered with Pertex® cover solution (PER 20000, medite GmbH, Burgdorf, Germany).

### Immunohistochemical KI-67 Staining

To assess the proliferative behavior of cells, magnetic hyperthermia treated (MF66) and untreated MDA-MB-231 tumor tissue was extracted 2 days and 28 days after the first magnetic hyperthermia treatment, with respect to the beginning of the study. 3 μm paraffin embedded tumor sections were stained for the presence of KI-67. Antigen retrieval was followed by a blocking step with avidin and biotin and a primary monoclonal anti-KI-67 antibody (Abcam, Cambridge, UK, 1:500 dilution) incubation step. A polyclonal goat anti-rabbit IgG (H + L)-Biotin-antibody (Dianova, Hamburg, Germany, 1:2,250 dilution) served as secondary antibody. For detection, a streptavidin-AP conjugate (Southern Biotech, Birmingham, USA, 1:75 dilution) and the REAL™ Detection System Alkaline Phosphatase/RED (DAKO, Glostrup, DK) were used. The slides were then stained with haematoxylin (Sigma-Aldrich, Steinheim, Germany) and covered with Faramount (DAKO, Glostrup, DK).

The evaluation of the stained slides was done by three blinded observers. Therefore KI-67 positive areas on whole tumor sections were viewed and grouped in distinctive categories: (I) absent proliferation (0% KI-67 positive area), (II) mild proliferation (>0–25% KI-67 positive area), (III) moderate proliferation (>25–50% of KI-67 positive area), (IV) high proliferation (>50–75% KI-67 positive area) and (V) very high proliferation (>75% KI-67 positive area).

### Statistics

We assessed the significance of tumor volume differences in the treatment groups by using the Mann–Whitney-*U*-Test (SPSS 20.0). P-values of *p* ≤ 0.05 and *p* ≤ 0.01 were chosen to be significant.

## RESULTS

### Magnetic Nanocrystal Synthesis and Characterization

**Fig. 2:**
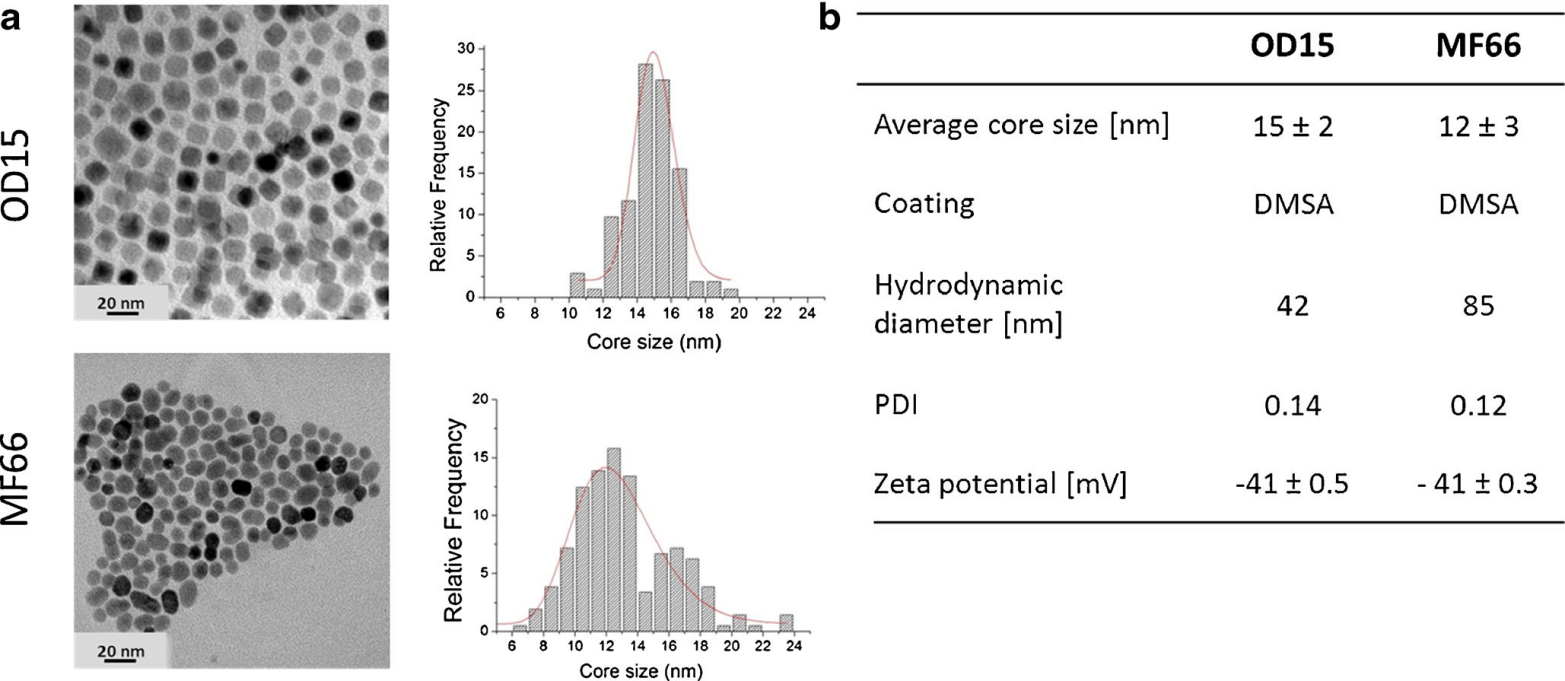
MNP characterization. (**a**) TEM micrographs and size distribution. Data was fitted to a lognormal distribution (*red line*). (**b**) MNP characteristics including average core size, coating, hydrodynamic diameter (z-average), Polydispersity index (PDI) and zeta potential of OD15 and MF 66. *MNP* magnetic nanoparticles.

MNP with average core size of 15 ± 2 nm and 12 ± 3 nm were obtained by a decomposition method in organic media (OD15) and *via* the co-precipitation route (MF66), respectively. Both MNP formulations showed a narrow size distribution (PDI: 0.14 for OD15, 0.12 for MF66) as it can be seen from TEM micrographs (Fig. 2a). Their hydrodynamic diameter in water is smaller for OD15 in comparison to MF66 as shown in Fig. 2b. Both MNP showed a comparable negative zeta potenial of about −41 mV.

**Fig. 3:**
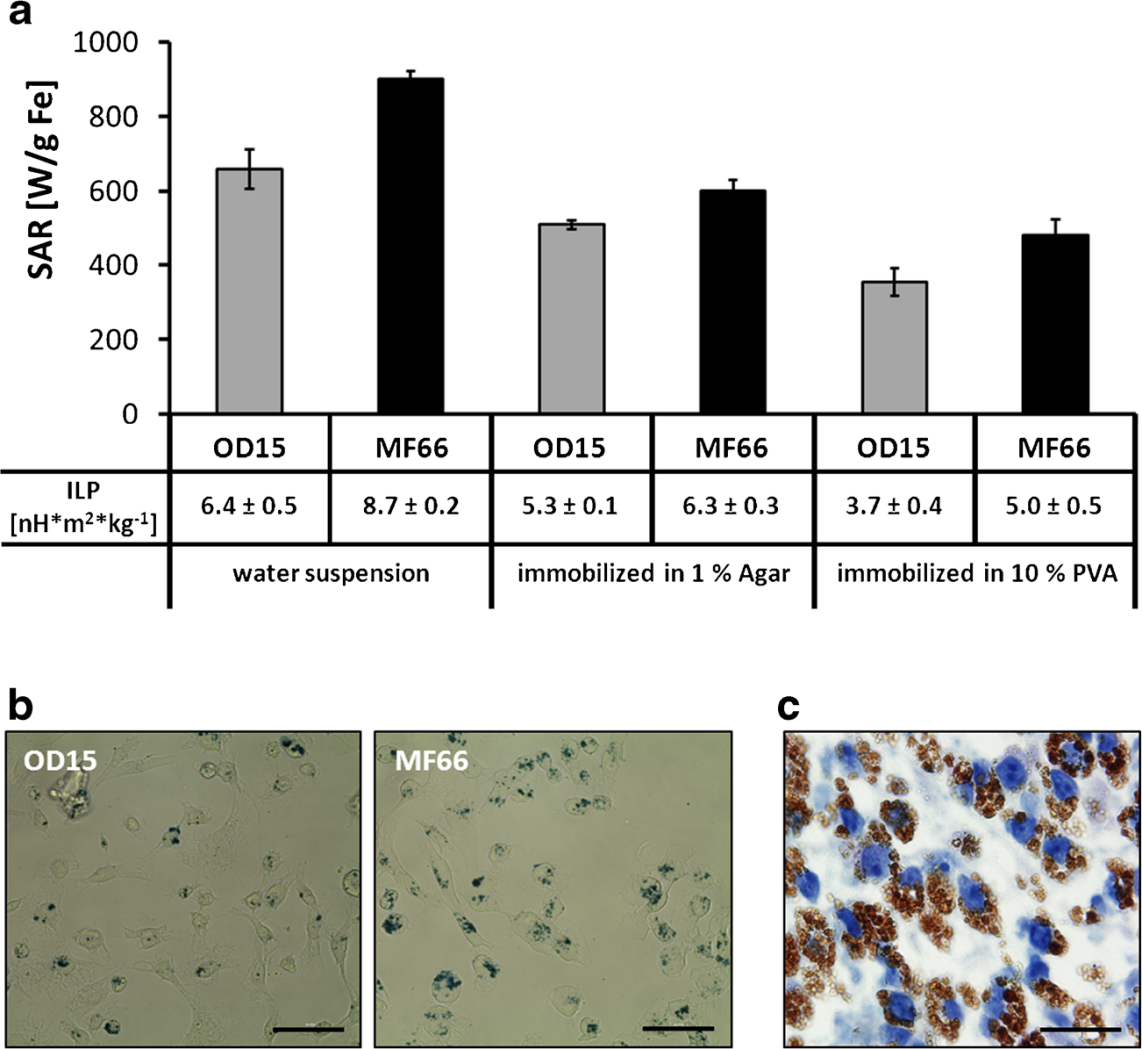
High SAR values for OD15 and MF66 MNP dispersed in water, agarose and polyvinylalcohol. The two latest reproduce immobilization conditions *in vivo*. (**a**) SAR values of both used MNP depending on the viscosity of the media (water, 1% (w/v) agar in water, 10% PVA in DMSO: water (80: 20% (v/v))). Intrinsic loss power (ILP) of OD15 and MF66 dispersed in water, agarose, and PVA. (**b**) Prussian Blue staining of MDA-MB-231 cells after incubation with 125 pg Fe/cell of OD15 and MF66 for 24 h at 37°C. *Bars*: 50 μm. (**c**) Hematoxylin stained tumor tissue of MF66 treated MDA-MB-231 xenografts 3 days after intratumoral application. Nuclei stained *blue*, MNP stained *brown. Bar*: 20 μm. *Error bars* indicate standard deviations. *MNP* magnetic nanoparticles, *PVA* polyvinylalcohol hydrogel, *SAR* specific absorption rate.

The exposure of MNP in a concentration of 2 mg Fe*ml^−1^ dispersed in water to the AMF (*H* = 15.4 kA*m^−1^, *f* = 435 kHz) resulted in distinctly high SAR values (658 ± 53 W*g_Fe_^−1^ for OD15, 900 ± 22 W*g_Fe_^−1^ for MF66). The minimization of Brownian motion by immobilization in agarose gels resulted in decreased SAR values (550 ± 14 W*g_Fe_^−1^ (OD15) and 650 ± 31 W*g_Fe_^−1^ (MF66), Fig. 3a). Immobilization in polyvinylalcohol hydrogels (PVA) led to an even stronger decrease (382 ± 39 W*g_Fe_^−1^ (OD15) and 520 ± 46 W*g_Fe_^−1^ (MF66)). The conversion of the obtained SAR values into intrinsic loss power (ILP) (Fig. 3a) led to values of 6.4 ± 0.5 nH*m^2^*kg^−1^ (OD15) and 8.7 ± 0.2 nH*m^2^*kg^−1^ (MF66) for MNP dispersed in water. Immobilization of nanoparticles in agar resulted in decreased ILP values: 5.3 ± 0.1 nH*m^2^*kg^−1^ for OD15 and 6.3 ± 0.3 nH*m^2^*kg^−1^ for MF66. Immobilization in PVA led to ILP values of 3.7 ± 0.4 nH*m^2^*kg^−1^ for OD15 and 5.0 ± 0.5 nH*m^2^*kg^−1^ for MF66.

Further on we demonstrated that after incubation of cells with OD15 or MF66, the MNP appeared in spots of high density in a perinuclear localization (Fig. 3b), indicating their cellular internalization. The localization of MNPs on the cell surface or within intercellular spaces could be excluded through thoroughly washing with HBSS. A comparable intracellular uptake could be seen in tumor tissue 3 days after intratumoral injection of MF66 into MDA-MB-231 tumors (Fig. 3c).

### Therapeutic Effects of *In Vivo* Magnetic Hyperthermia

**Fig. 4:**
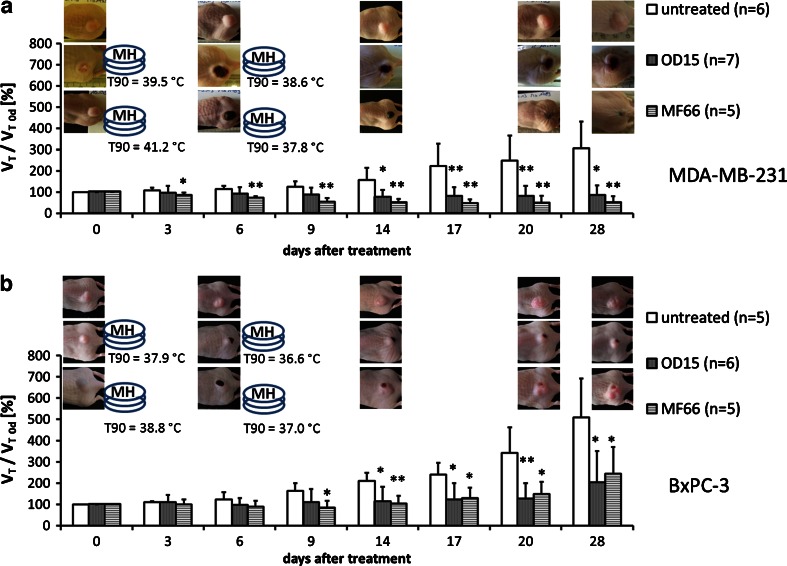
Repeated treatment of MNP bearing tumors in the AMF (*H* = 15.4 kA*m^−1^, *f* = 435 kHz) led to significant reduction of tumor volume compared to untreated ones. Effect of magnetic hyperthermia on the relative tumor volume in the course of a 4 week period after therapy in comparison to untreated tumors for MDA-MB-231 (**a**) and BxPC-3 xenografts (**b**). Additionally T90 temperatures and macroscopic tumor images are displayed. (* *p* ≤ 0.05, ** *p* ≤ 0.01 (Mann–Whitney-*U*-Test: treated *vs.* untreated)). *Error bars* indicate standard deviations. *AMF* alternating magnetic field, *MNP* magnetic nanoparticles, *T90* temperature exceeded by 90% of the tumor surface.

Our analyses showed that intratumorally injected MNP remain during the whole experimental period within the tumor site and are not transported to other organs (supplementary Figure S1). These findings suggested the possibility of repeated treatments of MNP bearing tumors (Fig. 1) in the AMF (*H* = 15.4 kA*m^−1^, *f* = 435 kHz), which led to an evident reduction of the tumor volume compared to untreated ones. Over a time period of 4 weeks, breast MDA-MB-231 tumors showed a significant shrinking of their relative volumes to 85% (OD15, *p* ≤ 0.05) and 50% (MF66, *p* ≤ 0.01) in relation to the tumor volumes before treatment. In contrast, untreated MDA-MB-231 tumors showed linear growth over the same time period, resulting in a relative tumor volume of 305% at day 28 in comparison to day 0. Magnetic hyperthermia treated tumors had, compared to untreated ones, a volume of 27% (OD15) and 17% (MF66), respectively (day 28 post first magnetic hyperthermia therapy, see Fig. 4a and Table I).

**Table I:** Parameters of Therapeutic Effects of *In Vivo* Magnetic Hyperthermia

		MDA-MB-231		BxPC-3	
		OD15	MF66	OD15	MF66
Applied magnetic material [mg Fe/100 mm^3^]		0.535	0.240	0.226	0.087
T90 [°C]	1st MH	39.5 ± 0.9	41.2 ± 1.7	37.9 ± 2.2	38.8 ± 0.9
	2nd MH	38.6 ± 1.4	37.8 ± 2.2	36.6 ± 1.8	37.0 ± 3.0
CEM43T90 [min]		1.5 ± 1.3	24.9 ± 30.2	1.9 ± 4.0	1.0 ± 1.0
Tumor volume growth rate		4.5 ± 2.4%/day	4.5 ± 2.4%/day	8.3 ± 3.4%/day	8.3 ± 3.4%/day
V_*T MH-28d*_/V_*T MH-0d*_		84.5 ± 46.3%	49.8 ± 29.2%	204 ± 146%	244 ± 124%
V_*T MH-28d*_/V_*T 28d*_		27.2%	16.6%	40.2%	48.1%

Applied magnetic materials, T90 temperatures, CEM43T90 values, tumor volume growth rates of untreated tumors and relative tumor volumes for OD15 and MF66 in breast cancer (MDA-MB-231) and pancreatic cancer (BxPC-3) xenografts are shown. V_T MH-28d_/V_T MH-0d_ : Quotient of mean tumor volume of magnetic hyperthermia treated animals at day 28 and mean tumor volume of magnetic hyperthermia treated animals at day 0; V_T MH-28d_/V_T 28d_: Quotient of mean tumor volume of magnetic hyperthermia treated animals at day 28 and mean tumor volume of untreated animals at day 28

*CEM43T90* cumulative equivalent minutes at a T90 temperature of 43°C, *MH* magnetic hyperthermia, *T90* temperature exceeded by 90% of the tumor surface

In pancreatic BxPC-3 xenografts, tumor growth was also significantly reduced after magnetic hyperthermia. The relative tumor volume at 28 days post first magnetic hyperthermia was 204% (OD15, *p* ≤ 0.05), 244% (MF66, *p* ≤ 0.05) compared to 507% of the untreated animal group. In relation to untreated tumors, magnetic hyperthermia resulted in a tumor volume of 40–50% (OD15 and MF66) compared to untreated tumors (*p* ≤ 0.05, day 28 post first magnetic hyperthermia, Fig. 4b, Table I).

In MDA-MB-231 tumors the growth arrest (<100% of initial tumor volume) persisted until 3 weeks after the second therapy, whereas in BxPC-3-xenografts a regrowth 1 week after the second magnetic hyperthermia was observed, which was stronger in the outer regions or rims of the tumors. The strongest volume increase took place in the last week between day 20 and 28 post first magnetic hyperthermia (Fig. 4). In this regard, *in vitro* thermal studies raising the temperature in the incubator showed similar results, where MDA-MB-231 cancer cells were more sensitive to heat than BxPC-3 cells (supplementary Figure S2).

During treatment, T90 temperatures (tumor surface temperature which is exceeded by 90% of the tumor area, see MATERIALS AND METHODS) were generally higher for MF66 than in OD15 injected tumors (MDA-MB-231 xenografts: MF66: 41.2°C, OD15: 39.5°C; BxPC-3 xenografts: MF66: 38.8°C OD15: 37.0°C, first magnetic hyperthermia, Table I). Macroscopically, the thermal response was accompanied by local changes on the tumor surface. The development of eschars took place, followed by emarginations and tissue loss. Due to the rather inhomogeneous MNP distribution after intratumoral application a regrowth of tumor tissue (see above) around former treated areas was observed between 14 and 28 days after first MNP or magnetic hyperthermia treatment.

**Fig. 5:**
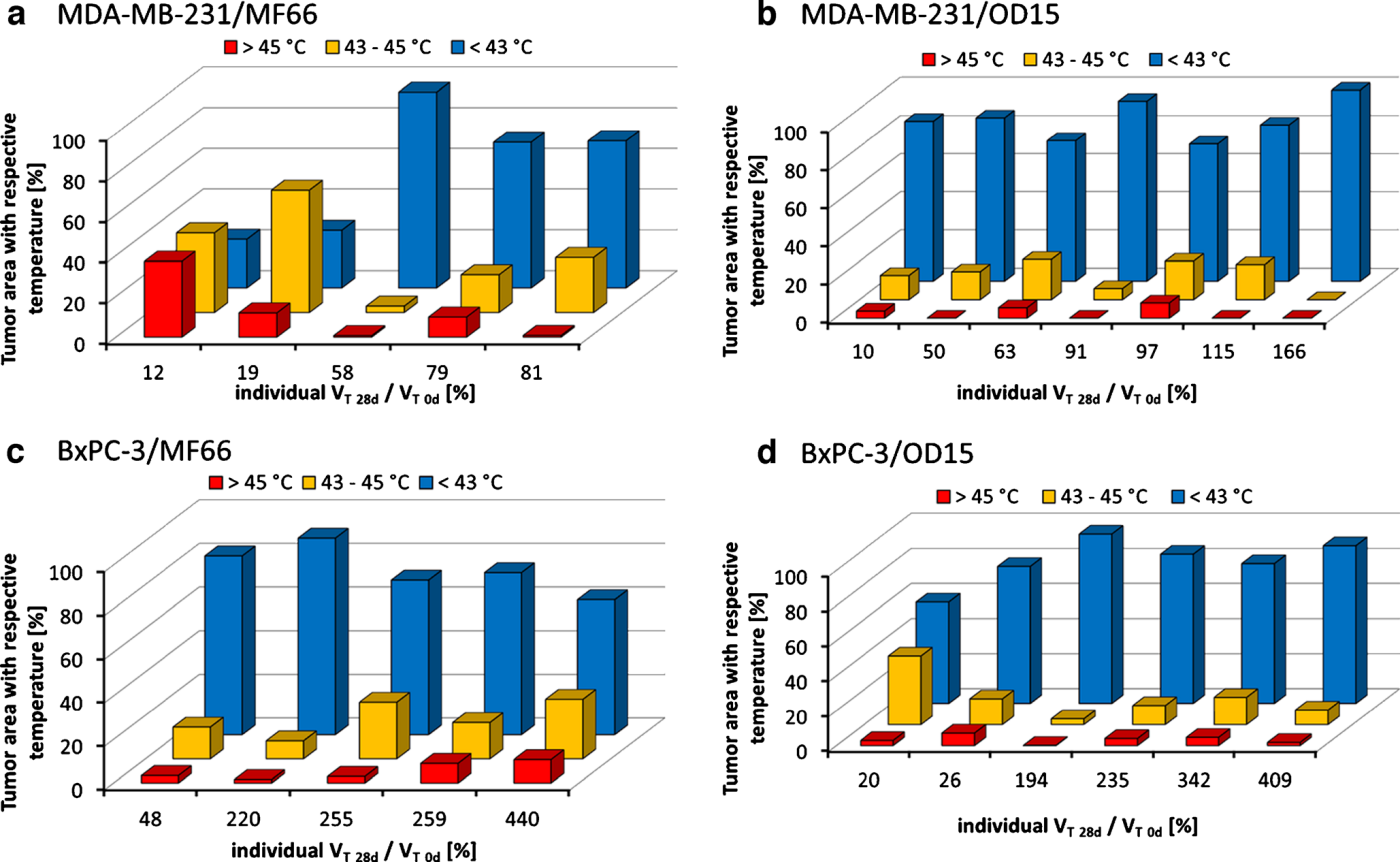
Despite the prevailing of tumor surface temperatures below 43°C, high temperature spots were also present. Temperature distribution maps (heat maps) of tumor surface temperatures of the two consecutive AMF treatments. The percentages of tumor area treated with temperatures below 43°C, between 43 and 45°C and above 45°C plotted against the individual relative tumor volume at 28 days after the first therapy are shown. MDA-MB-231 xenografts treated with magnetic hyperthermia using MF66 (**a**) or OD15 (**b**). BxPC-3 xenografts treated with magnetic hyperthermia using MF66 (**c**) or OD15 (**d**). *AMF* alternating magnetic field.

Interestingly, successful tumor therapy was achieved with comparatively low values of CEM43T90 (cumulative equivalent minutes at 43°C, see MATERIALS AND METHODS). They ranged between 1.5 min (OD15) and 24.9 min (MF66) for MDA-MB-231 and 1.9 min (OD15) and 1.0 min (MF66) for BxPC-3 (Table I). However, we could show that, although CEM43T90 values were below the hyperthermic effective duration of 60 min, a distinct reduction of tumor volumes still occurred. Heat map analysis revealed that localized heat spots were present in the tumor region with temperatures > 43°C (Fig. 5). Magnetic hyperthermia with MF66 MNP (Fig. 5a, c) induced an increased proportion of high temperature areas (*i.e.* the categories II (T 43–45°C) and III (T > 45°C), see MATERIALS AND METHODS) than OD15 MNP (Fig. 5b, d) (see also DISCUSSION). Overall the hyperthermic spots were larger in MDA-MB-231 than in BxPC-3 xenografts.

**Fig. 6:**
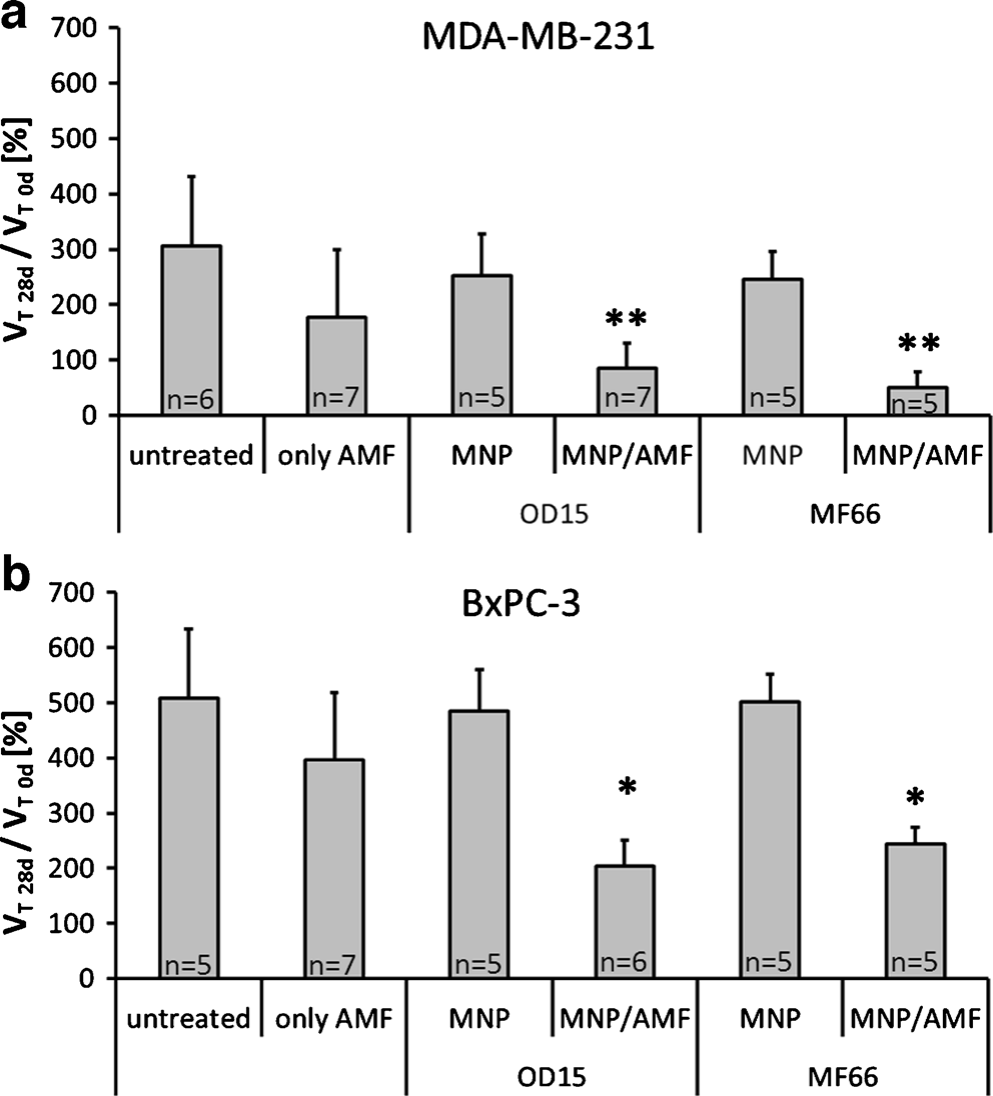
Neither the treatment within the alternating magnetic field (AMF, *H* = 15.4 kA*m^−1^, *f* = 435 kHz), nor with the magnetic nanoparticles (MNP, ≤0.5 mg Fe per 100 mm^3^) alone had a significant effect on tumor growth compared to the untreated animals. Relative tumor volume on day 28 of MDA-MB-231 (**a**) and BxPC-3 xenografts (**b**) for animals after only AMF or MNP treatment compared to untreated animals. Additionally, relative tumor volumes of magnetic hyperthermia treated animals at 28 days after first magnetic hyperthermia therapy for both xenograft models are displayed. (* *p* ≤ 0.05, ** *p* ≤ 0.01 (Mann–Whitney-*U*-Test: treated *vs.* untreated)). *Error bars* indicate standard deviations.

When tumors were treated with the AMF only (without MNP injection), there was no intratumoral temperature increase detectable (data not shown). The AMF treatment alone led to tumor volumes strongly overlapping with untreated tumors (Fig. 6). In this context, MDA-MB-231 xenografts tumors had 177 ± 123% of their starting volume (28 days after first magnetic hyperthermia) compared to 305 ± 126% of untreated tumors (28 days after first magnetic hyperthermia). In BxPC-3 pancreatic cancer xenografts, AMF treated tumors had 396 ± 165% of their initial volume in contrast to untreated tumors with 508 ± 185% (28 days after first magnetic hyperthermia).

MNP injection alone (without application of an AMF) showed a comparable growth of the tumors compared to untreated ones. Tumors of the MDA-MB-231 xenograft model elicited approximately 250% of their starting volume (28 days after treatment), BxPC-3 xenografts nearly 490% of their initial volume (28 days after treatment) for OD15 and MF66, respectively.

**Fig. 7:**
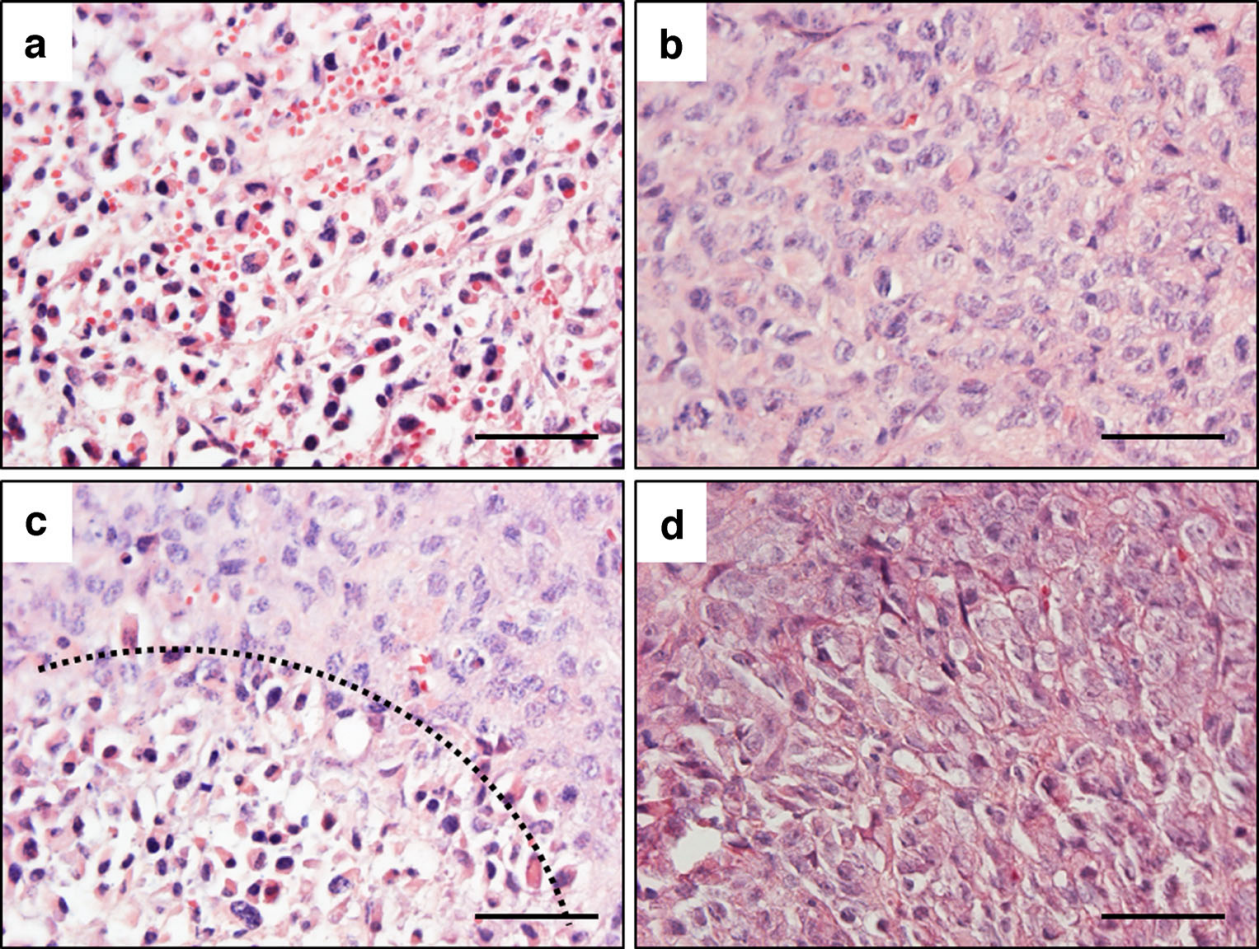
Magnetic hyperthermia leads to apoptosis and necrosis in tumor tissue. Hematoxylin/eosin stained tumor tissue of magnetic hyperthermia (MF66) treated and untreated MDA-MB-231 xenografts 2 days after first magnetic hyperthermia. (**a**) Necrotic and apoptotic tissue, (**b**) Vital tumor tissue, (**c**) Transition from necrotic and apoptotic to vital tumor tissue indicated by *dashed line*, (**d**) Vital untreated tumor tissue. Tissue sections were stained with hematoxylin and eosin. Nuclei stained *blue*, MNP stained *brown. Bars*: 50 μm. *MNP* magnetic nanoparticles.

Histological analysis of tumor sections of MDA-MB-231 xenografts at 2 days after the first magnetic hyperthermia (MF66) treatment revealed distinct alterations of tumor cell viability (Fig. 7). Magnetic hyperthermia induced extended regions of apoptotic and necrotic tumor tissue (Fig. 7a), even though vital areas were still present (Fig. 7b). Within the apoptotic and necrotic areas, the nuclei were condensed and fragmented, also cellular shrinkage took place. Between both areas clearly distinguishable margins were observed (Fig. 7c, dashed line). Untreated tumors consisted of viable, unaltered tissue without strong signs of apoptosis or necrosis (Fig. 7d).

**Fig. 8:**
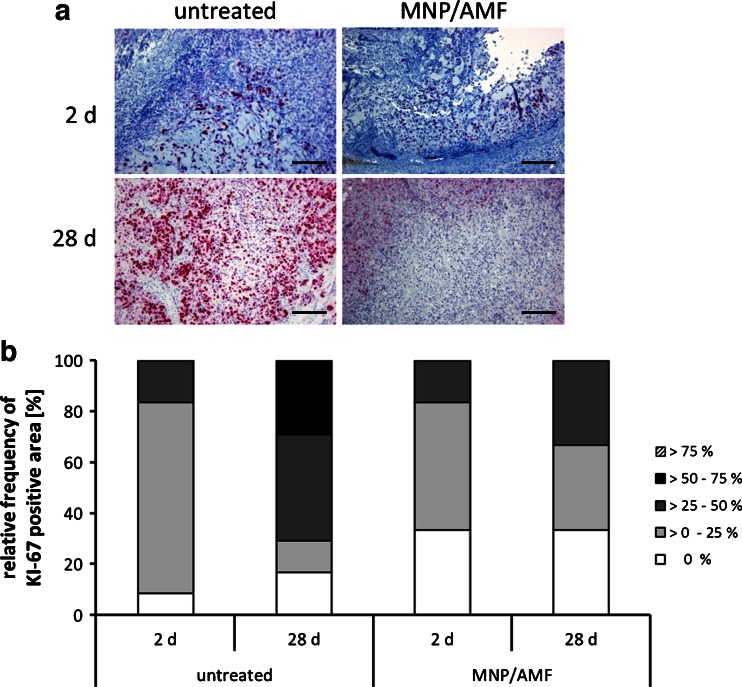
Magnetic hyperthermia diminished proliferative activity of tumor tissue compared to untreated tissue. (**a**) KI-67 staining of untreated and magnetic hyperthermia (MF66) treated MDA-MB-231 tumor tissue extracted 2 days and 28 days after the first magnetic hyperthermia. KI-67 positive cells stained *red*, nuclei stained *blue. Bars*: 200 μm. (**b**) Percentage of KI-67 positive tissue area 2 days and 28 days after the respective treatment. *Bars* represent categorical distribution of KI-67 positive area within the whole tissue section for all evaluated slides per group. *AMF* alternating magnetic field, *MNP* magnetic nanoparticles.

From the microscopical point of view, KI-67-staining was visible in the remaining vital areas of the tumors (Fig. 8a). Semi-quantitative examination of KI-67 staining as a marker of proliferation of tumors has shown marked differences between the different treatment groups. MDA-MB-231 xenografts treated with magnetic hyperthermia revealed a reduced proportion of areas with proliferating KI-67-positive cells at 2 days and 28 days after the first magnetic hyperthermia in comparison to the non-treated animal group (Fig. 8a, b).

**Fig. 9:**
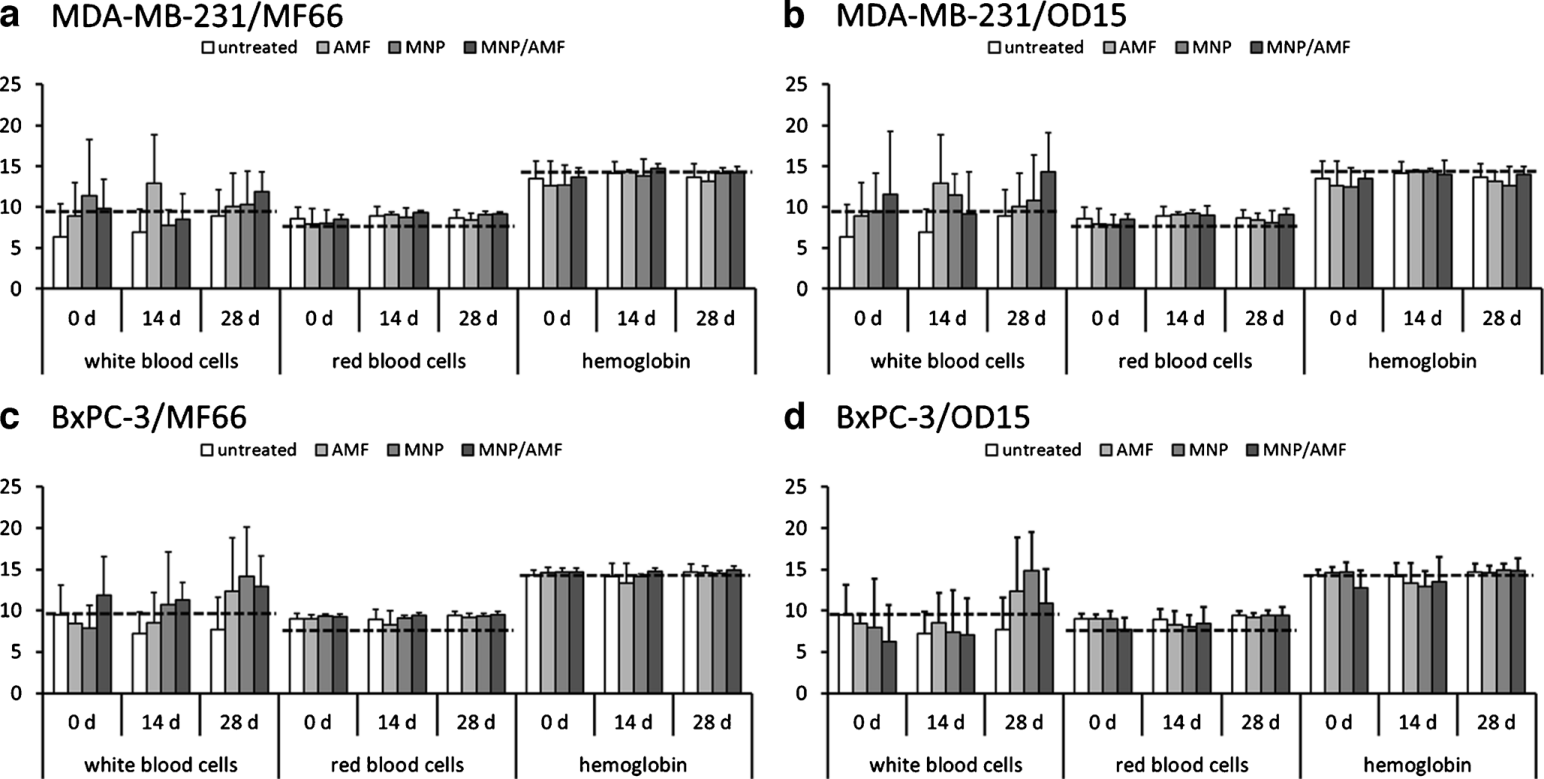
OD15 and MF66 magnetic hyperthermia treatment did not alter the blood composition, indicating a good biotolerability. The number of white blood cells (*10^3^*μl^−1^), red blood cells (*10^6^*μl^−1^), and the amount of hemoglobin (g*dl^−1^) are displayed for the magnetic hyperthermia treatment and the three control groups at 0 day, 14 days and 28 days after first hyperthermia. *Black dashed lines* refer to reference values (Harlan Laboratories, Venray, The Netherlands; http://www.harlan.com). MDA-MB-231 xenografts treated with MF66 (**a**) or OD15 (**b**). BxPC-3 xenografts treated with MF66 (**c**) or OD15 (**d**). *Error bars* indicate standard deviations. *AMF* alternating magnetic field, *MNP* magnetic nanoparticles.

Assessing the biocompatibility of the treatment modality, the sole intratumoral MNP injection, the exposure to the AMF only, and the combination of both (magnetic hyperthermia) did not result in any distinct changes of red and white blood cell count and hemoglobin concentration. The observed values were within the normal range values reported by a large provider of animal research models (Fig. 9) (Harlan Laboratories, Venray, The Netherlands; http://www.harlan.com). The body weight of the animals was also not affected by the presence of the MNP and the treatment in the AMF throughout the observation period of 28 days (supplementary Figure S3).

## DISCUSSION

We have studied the magnetic heating effect of two types of DMSA coated iron oxide nanoparticles with high SAR values on breast and pancreatic cancer xenografts in athymic nude mice. Hyperthermia treatment led to lethal temperatures within the tumors and resulted in a pronounced reduction of tumor volume compared to untreated tumors, with a stronger response in the slower growing breast cancer tumors. We carefully monitored the tumor surface temperature and were able to assess heterogeneous temperature distributions. The resulting temperature dosages of 1–24 CEM43T90 were lower than recommended in previous studies but showed great treatment efficiency in our study. Both particle types were biocompatible and did not affect the animal’s health without hyperthermia treatment.

### Heating Potential of the MNP

The MNP used in this study showed an exceptionally high heating potential after suspension in water. The ILP values obtained for the studied MNP are among the highest values ever reported on chemically synthesized iron oxide nanoparticles. The intrinsic loss power of 6.4 ± 0.5 nH*m^2^*kg^−1^ (OD15) and 8.7 ± 0.2 nH*m^2^*kg^−1^ (MF66) of the MNP suspended in water was more than twice as high as *e.g.* the values reported for 16 commercial superparamagnetic particles of comparable hydrodynamic sizes, which did not exceed 3.12 nH*m^2^*kg^−1^ (19). Cervadoro *et al.* also extensively reviewed ILP values of MNP available in the literature, mostly for frequencies around 500 kHz and reported predominantly ILP values below 1 nH*m^2^*kg^−1^, with some exceptions reaching ILP values between 4-5 nH*m^2^*kg^-1^ (authors refer as 4,000–5,000 10^−12^*W*m^2^*s*A^−2^*kg^−1^) (21). This underlines that the heating potenial of the MNP in this work readily exceeded those reported for most conventional MNP (22). Expressing SAR values as ILP is a physical magnitude for comparing heating efficiencies obtained under different *H*_*AC*_ conditions (19). The conversion of SAR into ILP values has proper sense only within the limits of the linear response theory where the magnetic response of MNP is linear with the applied magnetic field (*i.e.* when the maximum applied field amplitude is much smaller than magnetic field at which magnetization saturates) (23). Analysis of saturation magnetization of OD15 and MF66 at room temperature under quasi-static conditions resulted in field amplitude values of around 400 kA*m^−1^, which are approximately 20 times larger than the AMF amplitude employed in this study. Under AMF conditions at 435 kHz, the field amplitudes at which magnetization saturates are probably even larger. Therefore the AMF conditions employed in this work met the requirements for the conversion of SAR into ILP values.

Regarding the SAR and ILP data both synthetized formulations are suitable candidates for applications in magnetic hyperthermia.

The use of ILP would be desirable in future studies for establishing a heating efficiency parameter independent of AMF conditions. SAR values cannot be compared directly since it tightly depends on the field frequency and amplitude employed. On the other hand, when comparing the SAR values of the studied MNP in this work with others, their stronger heat release becomes apparent. OD15 and MF66 show SAR values in water between 658 and 900 W*g_Fe_^−1^ (*H* = 15.5 kA*m^−1^, *f* = 435 kHz) which are higher in comparison to values of 471 W*g_Fe_^−1^ reported by Yu *et al.* 2012 for 15 nm Zn_0.4_Fe_2.6_O_4_ MNP in more extreme AMF conditions (*H* = 37.4 kA*m^−1^, *f* = 500 kHz) (24). Other studies which applied similar AMF conditions (*H* = 11 kA*m^−1^, *f* = 411 kHz) to ours on MNP between 15 and 18 nm core size reported SAR values in the order of 500 W*g_Fe_^−1^ (25,26).

However, the heating potential of MNP dispersed in water can be different to the treatment situation in living tissue, because MNP were found to be immobilized to membranes or in intracellular vesicles (27,28). We showed that after immobilization, where one of the heating mechanims—Brownian motion—is inhibited, ILP values were still higher than those for water nanoparticle dispersions as reported by Kallumadil *et al.* 2009 (19).

The differences in particle size and size distribution, although small, led to differences in heating potential of the MNP, where the MF66 MNP showed a higher SAR than OD15 MNP.

The exceptionally high SAR and ILP values for the studied MNP are related to distinct reasons. On one hand, the chemical route of OD15 particles was optimized to obtain monodisperse nanoparticles exhibiting optimal magnetic properties and high SAR values (14). On the other hand, MF66 were synthesized by co-precipitation route resulting in non-uniform size and morphology MNP (Fig. 2a). Thus, the heating efficiency of MF66 may profit from the larger MNP size contribution and also from the elongated shape leading to higher anisotropy values, and therefore, higher SAR values (29).

After artificial immobilization, the SAR values of MF66 MNP were still higher than for OD15 MNP, supporting that their heating depended less on Brownian motion than OD15 MNP. Therefore the importance of Neél relaxation as predominating heating mechanism for the MNP is underlined (30). In this regard, particularly MF66 MNP are favorable for *in vivo* applications for controlling the heat exposure.

### Therapeutic Effects of *In Vivo* Magnetic Hyperthermia

We could show that magnetic particle induced hyperthermia significantly reduced (MDA-MB-231) or inhibited (BxPC-3) tumor growth compared to untreated tumors. Successful magnetic hyperthermia could already be achieved by 1–2 CEM43T90, although a higher temperature dose led to further decrease of the tumor volume. Nevertheless, a comparison of these values to the existing literature is challenging, because of the variety of methods used to deliver hyperthermia and to monitor thermal dose and due to the fact that most groups have been treating the tumors not only with hyperthermia but also with radiotherapy.

However, to the best of our knowledge, the CEM43T90 temperatures needed to achieve these effects were lower than ever reported in the literature. A number of studies which report CEM43T90 temperatures fail to find a correlation between thermal dosage of above 10 CEM43T90 and tumor response (8,9), whereas other studies, which compared low temperature doses (lower than 5 CEM43T90) with high ones (10–100 CEM43T90) reported low therapy response for particularly the low dose hyperthermia (12,13).

Presumably, the observed high efficiency of comparatively low CEM43T90 is, at least in part, caused by the nature of MNP induced hyperthermia, which was reported to be more cytotoxic to tumor cells than hot water hyperthermia (31,32). The underlying reasons can be found in the occurrence of hyperthermic spots, which we were able to identify by infrared thermography. This refers to very localized areas of the tumors that reached temperatures between 43 and 45°C (hyperthermic temperatures) and above 45°C (mild thermoablative temperatures). Emanating from these spots, intratumoral temperature gradients might well lead to cell death up to a defined distance from the magnetic material. These heating spots are the result of the intratumoral distribution pattern of the magnetic material.

It is worth mentioning that the calculation of T90 and CEM43T90 values was performed using data inferred from tumor surface temperatures (supplementary Figure S4). Taking into account that non-invasive thermometry is not available at the present, we were able to show that the temperatures inside the tumor were even higher (0.1–0.2°C) than temperatures measured at the tumor surface (supplementary Figure S5) for a BxPC-3 xenograft injected with MNP and treated within the AMF. The acquisition of the temperature data used in this study was suitable to address the specific questions, however T90 and CEM43T90 values within the tumor region could be slightly higher than the ones obtained at the tumor surface.

Obviously, even though mostly of heterogeneous nature, the intratumoral deposition of magnetic material is highly efficient as our results show (33,34). The accumulation of nanoparticles in sufficient amounts in tumor tissue after systemic (intravenous) application is a yet unsolved challenge in magnetic hyperthermia research (21,35). Until the major challenges such as the nanoparticle removal from the blood stream and an enhanced tumor uptake and retention are resolved, intratumoral injection is the best method to reach a sufficient amount of nanoparticles at the tumor site to induce heating (35,36). Furthermore, intratumoral application of magnetic material is readily applicable in the clinical setting using stereotactical methods in radiology, where the magnetic material can *e.g.* be applied during a biopsy process under visual guidance.

A distinct reduction of the tumor volumes was observed, even though tumors did not disappear completely after magnetic hyperthermia therapy. Interestingly, it has been shown that less aggressive therapeutic approaches can be beneficial for tumor regression compared to more radical approaches leading to hypoxic areas by destroying tumor vasculature (37). Hypoxia is known to select for more malignant cells with increased metastatic potential (38). It remains to be elucidated how the corresponding intratumoral particle distribution patterns can cover the whole target area with adequate temperature dosages in order to get the tumor growth under control.

Magnetic hyperthermia effects in both tumor models were provoked by a smaller amount of MF66 MNP than OD15 MNP. Since MF66 MNP had a higher ILP than OD15, and, in consequence, smaller amounts are required at the tumor, we conclude that ILP of the magnetic material has a higher impact on therapy outcome than the amount of magnetic material applied to the tumor. In perspective of systemic applications small MNP amounts at the tumor site is desirable for hyperthermia treatment, since delivery to the tumor is, despite of possible nanoparticle functionalizations with target-affine ligands, unalterably coupled to the extent of the enhanced permeability and retention effect (EPR effect) (35,39).

Independently of the MNP type (MF66 or OD15), the applied MNP amount or the temperature dosage used for magnetic hyperthermia, the therapeutic effects were cell line dependent in a wide sense. For example, MDA-MB-231 tumors treated with OD15 received a temperature dosage intermediate between OD15 treated BxPC-3 and MF66 treated BxPC-3, while the amount of applied magnetic material was higher for MDA-MB-231 xenografts. The different dosages were the result of the varying interstitial pressure between these tumors (lower values for MDA-MB-231compared to BxPC-3, own observations). However, tumor volume of MDA-MB-231 tumors was distinctly smaller at the end of the experiment than that of BxPC-3 tumors. Whether this difference was caused by a higher proliferation rate of BxPC-3 cells (BxPC-3 doubling time *in vitro*: 1.5 ± 0.5 days *vs*. 1.8 ± 0.5 days for MDA-MB-231) or by differences in thermotolerance between the two cell lines and/or by the effect of tumor interstitial pressure on magnetic material dosing will be the subject of further in depth investigations. First *in vitro* thermal studies indicated that MDA-MB-231 are more sensitive to heat than BxPC-3 cells and are therefore more effectively inactivated by hyperthermia treatment (supplementary Figure S2), which is in line with our *in vivo* observations. Comparing the first and second hyperthermia treatment it is noticeable that T90 temperatures are always lower during the second therapy. The reason for this observation is primarily not in MNP degradation, but rather the massive tissue remodeling which takes place after the first treatment leading to MNP redistribution. Previous works (40) have shown that the heating potential of MNPs after intratumoral application is not altered for several days and therefore the heating efficiency is not influenced. The appearance of eschars and disintegration of dead tissue results in dense intratumoral MNP clusters leading to high temperature spots. In order to not excess the hyperthermic temperature, AMF power often had to be reduced during the second treatment, leading to an overall lower temperature dose.

Importantly, we could demonstrate each magnetic hyperthermia component alone, either AMF or MNP exposure, did not lead to significant effects on tumor volume, underlining that the therapeutic effects depended merely on the combination of both the MNP and the AMF treatment (and the hyperthermic temperatures induced by the magnetic hyperthermia). Hence, nanoparticle application without AMF does not transmit cytotoxic effects and neither does AMF exposure when no magnetic material is present.

As shown for the MDA-MB-231 xenograft model, tumor volume of the magnetic hyperthermia group stagnated in the course of the long term observation during 28 days, whereas the untreated control group showed continued linear tumor growth. These observations were in accordance with the KI-67 expression pattern that predominantly showed KI-67 expression in 0 to 25% of tumor area after the first AMF treatment and at the end of the experimental period on day 28. The untreated and MNP only group on the other side, showed an increase in KI-67 expression from day 2 to day 28 with KI-67 expression in larger areas of the tumors. Hence, magnetic hyperthermia depleted the proliferative activity of treated tumors, which was macroscopically observed as tumor volume reduction and growth stagnation. Furthermore, the low proliferative activity even 21 days after the last magnetic hyperthermia leads us to the conclusion that, in this case, tumor volume is unlikely to strongly increase over time.

In the view of further investigations towards clinical studies involving the MNP used in the present study, it is important to announce that they showed excellent biocompatibility. Animals showed no affection throughout the experiments and we observed no pathological change in the white and red blood cells and hemoglobin levels. Body weight of the animals was also not affected (supplementary Figure S3).

In the future, a systemic application instead intratumoral MNP application would be desirable to be able to treat deep seated tumors and metastases. Importantly it has to be kept in mind that by functionalizing nanoparticles with targeted molecules only the uptake into tumor or endothelial cells can be improved, whereas the delivery of MNP from the vascular system into the tumor interstitium depends solely on the EPR effect, independently of the nanoparticle functionalization (35). In contrast, after intratumoral application defined amounts of iron is deposited, which generally remain at the tumor site, making hyperthermia treatment much more projectable and predictable.

## CONCLUSION

In conclusion, magnetic hyperthermia using iron oxide nanoparticles with exceptionally high ILP lead to an effective tumor growth reduction or delay in subcutaneous xenografts although measured CEM43T90 temperatures were lower than recommended in the literature. Both nanoparticle formulations were adequate for *in vivo* hyperthermia experiments, but MF66 (aqueous synthesis route) displayed a higher heating potential and led to higher T90 values than OD15 (organic synthesis route). The better response of breast cancer xenografts to the therapy compared to pancreatic tumors is the result of multifactorial reasons and will be subject of further investigations. Furthermore the excellent biocompatibility of the MNP and localized therapy made the therapy well tolerable for the animals.

## Electronic supplementary material

Below is the link to the electronic supplementary material.

ESM 1 (DOCX 1334 kb)

## ABBREVIATIONS

AMF: Alternating magnetic field
CEM43: Cumulative equivalent minutes at 43°C
CEM43T90: Cumulative equivalent minutes at a T90 temperature of 43°C
ILP: Intrinsic loss power
MNP: Magnetic nanoparticles
SAR: Specific absorption rate
T90: Temperature exceeded by 90% of the tumor surface
