# Supplementary material for: High Therapeutic Efficiency of Magnetic Hyperthermia in Xenograft Models Achieved with Moderate Temperature Dosages in the Tumor Area
Source: Pharm Res. 2014 Jun 3;31(12):3274–88. doi: 10.1007/s11095-014-1417-0 (PMC4224751; doi:10.1007/s11095-014-1417-0)
Supplement: Supplementary file 1 — (DOCX 1334 kb) [file 11095_2014_1417_MOESM1_ESM.docx]

Supporting Information

High Therapeutic Efficiency of Magnetic Hyperthermia in Xenograft Models Achieved with Moderate Temperature Increases in the Tumor Area

*Susanne Kossatz^1*^, Robert Ludwig^1*^, Heidi Dähring^1^, Volker Ettelt^1^, Gabriella Rimkus^1^, Marzia Marciello^2^, Gorka Salas^2, 3^, Vijay Patel^4^, Francisco J. Teran^3, 5^, Ingrid Hilger^1^*

^*^ contributed equally

*^1^*Institute for Diagnostic and Interventional Radiology I, Jena University Hospital – Friedrich Schiller University Jena, Bachstraße 18, D-07740 Jena, Germany

*^2^*Instituto de Ciencia de Materiales de Madrid, ICMM-CSIC, Sor Juana Inés de la Cruz 3, Campus Universitario de Cantoblanco, 28049 Madrid, Spain

*^3^*Instituto Madrileño de Estudios Avanzados en Nanociencia, Campus Universitario de Cantoblanco, 28049 Madrid, Spain

*^4^*Liquids Research Limited, Deiniol Road, Bangor, Gwynedd, United Kingdom

*^5^*Unidad Asociada de Nanobiotecnología CNB-CSIC & IMDEA Nanociencia, Campus Universitario de Cantoblanco, 28049 Madrid, Spain

**Figure S1.**

Figure S1. Exogeneously applied iron could only be detected in tumors and not in other organs at a time point of 4 weeks after intratumoral MF66 injection. (A) In BxPC‑3 xenografts, MF66 related iron was detected by magnetic particle spectroscopy (see supplementary methods). (B) In MDA-MB-231 xenografts, whole iron content was detected using flame atomic absorption spectrometry (see supplementary methods). Error bars indicate standard deviations. DM: dry mass.

**Figure S2.**

**Figure S2. MDA-MB-231 cells are more sensitive to heat compared to BxPC-3 cells.** Cell viability measured by determining the NADH content normalized to non-treated controls (relative NADH content, see supplementary methods) of MDA-MB-231 (A) and BxPC-3 (B) cells at 1, 24, and 48 h after thermal treatment. Duration and treatment temperatures are given as cumulative equivalent minutes at 43 °C (CEM43). Error bars indicate standard errors.

**Figure S3.**

**Figure S3. OD15 and MF66 MNP or magnetic hyperthermia treatment did not alter the body weight.** Body weight of MDA-MB-231 and BxPC-3 xenografts treated with magnetic hyperthermia, only MNP, only AMF or untreated, monitored over a period of 28 days post the first magnetic hyperthermia, respectively the begin of the study. MDA-MB-231 xenografts treated with MF66 (A) or OD15 (B). BxPC-3 xenografts treated with MF66 (C) or OD15 (D). Error bars indicate standard deviations. AMF: alternating magnetic field, MNP: magnetic nanoparticles.

**Figure S4.**

Figure S4. Infrared thermographic images of BxPC-3 xenograft treated with MF66 during 1^st^ AMF treatment shows inhomogeneous temperature distribution and occurrence of local heat spots in the tumor region. Infrared thermographic images of a BxPC-3 xenograft injected with MF66 (0.087 mg Fe per 100 mm^3^) during the 1^st^ magnetic hyperthermia treatment. (A) Images were taken distally from the tumor using an infrared thermography camera. (B) Over each tumor a polygonal region of interest (ROI, white polygon) was placed in a size specific manner and the corresponding temperature data at 10, 30 and 50 min post onset of the AMF was extracted (see methods).

**Figure S5.**

Figure S5. Temperatures inside a tumor during magnetic hyperthermia are comparable or even higher than temperatures measured on the tumor surface.

(A) Temperature distribution during the first 30 minutes of magnetic hyperthermia determined using fiber optic temperature sensors located at different positions inside the tumor (red & green graphs) and on the tumor surface (black graph). (B) Positions and distances between the used fiber optic temperature sensors are depicted within a cross section of a schematic tumor.

**Supplementary methods:**

**alamarBlue^®^ Assay**

Cell viability was assessed as relative NADH content via the alamarBlue^®^ Assay (Invitrogen Corporation, Carlsbad, CA, USA). In this context 5,000 breast adenocarcinoma MDA-MB-231 and 5,000 pancreatic adenocarcinoma BxPC‑3 cells were seeded into a 96-well plate (Greiner Bio-One GmbH, Frickenhausen, Germany) 48 hours prior to the experiment (37 °C, 5 % CO_2_). For hyperthermia treatment, plates were exposed to a temperature dose of 90 cumulative equivalent minutes at 43 °C in an incubator. An incubator was used to model homogeneous temperature conditions. After a 24 hour postincubation period (37 °C, 5 % CO_2_), cells were washed three times with HBSS and then 10 % alamarBlue^®^ in culture medium was added. After 4 h at 37 °C and 5 % CO_2_ the fluorescence was measured at 530 ‑ 560 nm excitation wavelength and 590 nm emission wavelength using an Infinite M1000 PRO plate reader (Tecan Austria GmbH, Grödig, Austria). The determined NADH content after hyperthermia was normalized to values obtained for cells incubated at 37 °C. Experiments were done in triplicate with three parallels each.

**Iron Biodistribution**

We determined iron biodistribution at the end of the experiment (day 28) using two different methods to cross-validate the results. The main object was to determine if MNP are stably located at the tumor site or if organ deposition occurs. Here, we analysed animals bearing BxPC-3 and MDA-MB-231 xenografts that received MF66 MNP intratumorally (control: no MF66) which were not treated in the AMF. We used flame atomic absorption spectrometry (FAAS), which determines whole iron(II/III) oxide in organs and tumors, and magnetic particle spectroscopy (MPS), which measures only the iron that relates to the applied MNPs ([Gleich and Weizenecker 2005](#_ENREF_1)).

For FAAS, after animal dissection, tissues were partitioned into equally sized parts and dried at 40 °C for 48 h on a shaking incubator. After weighing, tissues were digested with 1 ml ashing solution (3:2 mixture of 65 % nitric acid (Suprapur, VWR) and 70 % perchloric acid (Suprapur, Merck Millipore)) and ashed by heating at 70 °C, 160 °C and 250 °C for 1 h each. The ash residue was dissolved in 1 ml 65 % nitric acid and iron content was measured with FAAS (AAS 5 FL, Analytik Jena). Iron content of the samples was determined using a calibration standard with known iron content (0, 5, 10, 20, 30 and 50 µmol Fe/l in 37 % hydrochloric acid (Roth). The iron content was calculated using the formula

*mg/g Fe DM = Fe [mmol/l] x dilution factor x molar mass Fe [g/mol]*

*DM [mg]*

Where *Fe [mmol/l]* is the result of FAAS measurement and *DM* (dry mass) is the weight of the dried tissue.

For MPS, dissected tissues were homogenized with a ball mill (Tissue Lyser, Qiagen, Hilden, Germany), transferred to 0.1 mL cups and weighed. Samples were measured using a 25 mT magnetic particle spectrometer (Bruker, Billerica, MA, USA). Each measurement took 10 s and was corrected for background. Lyophilized MF66 and OD15 were used as reference measurement.

**References:**

**Gleich, B. and J. Weizenecker (2005). "Tomographic imaging using the nonlinear response of magnetic particles." Nature 435(7046): 1214-1217.**
